# Exploratory Analysis of Early Renal Function Changes After Transcatheter Aortic Valve Implantation (TAVI): Limited Predictive Value Beyond Baseline Renal Function

**DOI:** 10.3390/jcm15103726

**Published:** 2026-05-12

**Authors:** Rosa Alba Pugliesi, Shu Fon Muna, Andreas H. Mahken, Nour Maalouf, Georgios Chatzis, Jonas Apitzsch

**Affiliations:** 1 Department of Biomedicine, Neuroscience and Advanced Diagnostics (BiND), University of Palermo, Via del Vespro 129, 90127 Palermo, Italy; 2 Department of Urology, University Hospital OWL, Evangelical Hospital Bethel gGmbH, Schildescher Str. 99, 33611 Bielefeld, Germany; shu-fon.muna@evkb.de; 3 Department of Diagnostic and Interventional Radiology, University Hospital of Marburg, 35043 Marburg, Germany; 4 Department of Diagnostic and Interventional Radiology, University Hospital of Tübingen, 72076 Tübingen, Germany; nourmaalouff@gmail.com; 5 Department of Cardiology, Angiology, and Intensive Care Medicine, University Hospital, Philipps University of Marburg, 35043 Marburg, Germany; chatzis@staff.uni-marburg.de; 6 Department of Radiology and Nuclear Medicine, Helios Hospital Pforzheim, 75175 Pforzheim, Germany; jonas.apitzsch@helios-gesundheit.de

**Keywords:** TAVI, renal function, creatinine, eGFR, contrast-induced nephropathy

## Abstract

**Background:** In elderly, multimorbid patients, renal function changes are frequent following transcatheter aortic valve implantation. However, the early renal recovery following the relief of aortic stenosis is not sufficiently characterized. **Methods:** This retrospective single-center study comprised 410 consecutive patients who underwent TAVI. Serum creatinine and estimated glomerular filtration rate (eGFR) were measured prior to and within 72 h of TAVI to evaluate renal function. Primary outcomes were defined as absolute alterations (Δcreatinine and ΔeGFR). Spearman’s correlation and multivariable regression were implemented to assess associations. **Results:** The mean age was 82.0 ± 8.7 years, and 46.9% of the participants were female. The eGFR demonstrated modest improvement (mean ΔeGFR +3.83 mL/min/1.73 m^2^), while creatinine showed minimal change (mean Δ −0.015 mg/dL). Renal function exhibited bidirectional alterations. Baseline creatinine was inversely associated with Δcreatinine (ρ = −0.127, *p* = 0.010), which was consistent with regression to the mean. Conversely, baseline eGFR was not associated with ΔeGFR (ρ = 0.004, *p* = 0.934). There were no significant correlations between renal changes and BMI (ρ = −0.041 and ρ = 0.047; both *p* > 0.30). In multivariable analysis, baseline creatinine remained independently associated with Δcreatinine (β = −0.279, *p* < 0.001), whereas ejection fraction exhibited a modest association (β = 0.012, *p* = 0.020). Acute kidney injury was observed in 13.9% of the population (57/410) and was not independently correlated with baseline variables. **Conclusions:** Early renal alterations following TAVI are frequent and frequently favorable; however, they are primarily indicative of baseline renal function, with limited independent predictive value of other variables. The results should be regarded as hypothesis-generating.

## 1. Introduction

Transcatheter aortic valve implantation (TAVI) has become the standard of care for patients with severe aortic stenosis who are at increased surgical risk, and its use has expanded steadily into intermediate- and lower-risk populations [1,2]. Despite these advances, the typical TAVI population remains characterized by advanced age, multiple comorbidities, and a high prevalence of chronic kidney disease [3]. Within this vulnerable clinical context, renal function represents a particularly fragile physiological domain, and even modest peri-procedural changes may have important prognostic and therapeutic implications [4].

Post-interventional renal dysfunction after TAVI is a well-recognized complication and has consistently been associated with prolonged hospitalization, higher short- and long-term mortality, and increased health care utilization [5]. Several mechanisms have been proposed to explain kidney injury in this setting, including contrast-induced nephrotoxicity, peri-procedural hypotension, embolic events, inflammatory responses, and exposure to nephrotoxic medications [6]. Consequently, renal deterioration is often conceptualized primarily as an adverse outcome to be prevented [7]. However, this perspective captures only part of the renal trajectory after TAVI [8]. Relief of severe aortic stenosis can improve cardiac output, renal perfusion, and venous congestion, thereby creating physiological conditions that may stabilize or even improve kidney function, particularly in patients with cardiorenal interactions driven by low-flow states [9].

Contrast exposure remains one of the most modifiable procedural factors influencing renal outcomes after TAVI [10]. Although total contrast volume has historically been employed to assess renal risk, increasing evidence indicates that absolute volumes may insufficiently capture individual susceptibility [11]. Patient-specific factors such as body weight, baseline renal function, and overall physiological reserve likely modulate the renal effects of contrast media [12]. Weight-adjusted contrast supplementation has thus become a potentially more significant metric; however, it remains insufficiently studied within TAVI-specific populations [13]. Furthermore, the majority of previous research has concentrated on the development of acute kidney injury characterized by categorical thresholds, rather than investigating renal function as a continuous and bidirectional process encompassing both decline and recovery [14].

This emphasis on isolated risk factors and binary outcomes highlights a significant gap in the existing evidence [15]. Renal responses after TAVI are variable, and a comprehensive evaluation that concurrently incorporates demographic, anthropometric, and procedural factors may provide a more detailed understanding of individuals at risk and those likely to gain renal benefit from the intervention [16]. In particular, protective factors associated with early post-procedural renal recovery have received relatively limited attention, despite their potential importance for patient counseling, risk evaluation, and personalized peri-procedural management [17].

In this context, the current work examines the clinical issue of post-TAVI renal function changes by carefully assessing short-term changes in serum creatinine and estimated glomerular filtration rate (eGFR) in a real-world population. Instead of limiting the analysis to renal deterioration alone, this study explicitly recognizes renal recovery as a common and clinically significant outcome. By examining changes in renal function within 72 h after TAVI, the study highlights the immediate association between procedural factors and the patient’s susceptibility or resilience.

In a previous analysis from our group, we specifically investigated the role of procedural and device-related characteristics, including valve platform and diameter, on early renal outcomes after TAVI [18]. In that study, which included 371 patients with complete covariate data, we found no independent association between valve type or size and post-procedural renal function changes or KDIGO-defined acute kidney injury. These findings suggest that structural and device-related factors may play a limited role in determining early renal trajectories compared with patient-specific and procedural variables. Building on this prior work, the present study shifts the focus toward identifying individualized risk and protective factors, with particular attention to contrast exposure and patient-related characteristics.

The main aim of this retrospective analysis is to determine patient-specific risk and protective factors related to alterations in renal function following TAVI. Specifically, the study aims to address two primary research questions: which demographic, anthropometric, and procedural characteristics are associated with deterioration of renal function after TAVI, and which factors are linked to renal improvement, as indicated by a reduction in serum creatinine or an increase in eGFR. We hypothesize that renal recovery after TAVI is common and that weight-adjusted contrast exposure, rather than absolute contrast volume, is more closely associated with short-term renal alterations. By adopting an integrative and patient-centered analytical approach, this study aims to contribute to a more balanced understanding of renal vulnerability and recovery after TAVI and to inform more personalized renal protection strategies in contemporary structural heart interventions.

## 2. Material and Methods

### 2.1. Study Design and Population

This study was conducted as a retrospective, exploratory observational analysis including all consecutive patients who underwent TAVI from January 2018 to December 2021 at a tertiary cardiovascular center.

Demographic and anthropometric variables included age, sex, height, weight, and body mass index (BMI). Procedural and imaging-related parameters comprised contrast volume administered during pre-procedural CT, contrast volume during the TAVI procedure, total contrast volume, and weight-adjusted contrast dose expressed as mL/kg. Additional clinical variables included valve/annulus diameter and baseline left ventricular ejection fraction (EF). Baseline renal function (pre-procedural creatinine and eGFR) was explicitly included in correlation and multivariable analyses to account for regression to the mean inherent in change-score outcomes.

All variables were extracted from standardized electronic medical records and procedural documentation to ensure consistency.

The study protocol was approved by the institutional review board (internal reference number: F-2022-006) in accordance with the Declaration of Helsinki and applicable institutional regulations. Written informed consent was obtained from all participants. Contrast volume data, which were not available in earlier analyses of this cohort, were retrospectively retrieved from procedural and imaging archives for the present study.

### 2.2. Inclusion and Exclusion Criteria

**Figure 1:**
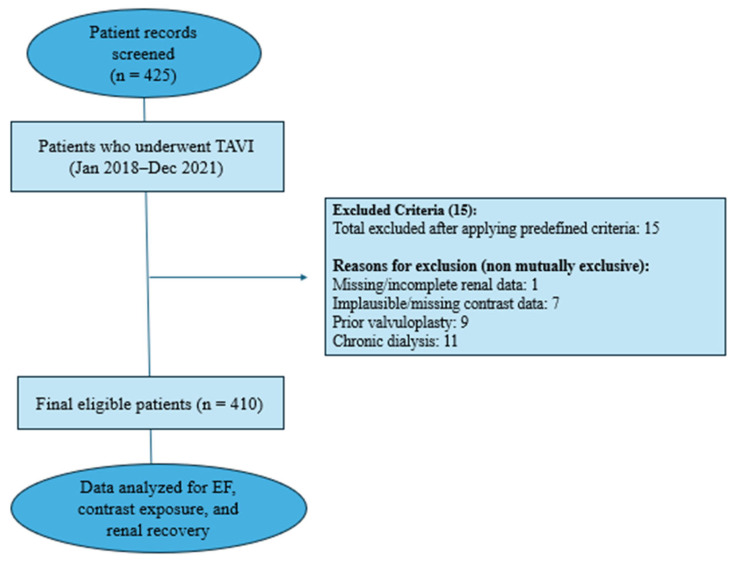
Study Flowchart. Overview of participant recruitment, interventions, data collection, and analysis process in the study.

A total of 425 patients who underwent TAVI during the study period were initially available for analysis. Predefined exclusion criteria were applied to ensure data integrity and clinical comparability (Figure 1).

These included missing or implausible contrast volume data (defined a priori as <25 mL), absence of documented dates for computed tomography (CT) imaging or the TAVI procedure, prior balloon or surgical aortic valvuloplasty, and pre-existing dependence on renal replacement therapy (including hemodialysis or peritoneal dialysis).

After applying these criteria, 410 patients with complete pre- and post-procedural renal biomarker measurements were eligible for the primary renal outcome analysis.

Eligibility for inclusion required successful completion of the TAVI procedure, availability of serum creatinine and estimated glomerular filtration rate (eGFR) measurements both before and after the intervention, and documented left ventricular ejection fraction (EF) as a key covariate.

Patients were excluded if renal biomarker data were incomplete, if contrast medium volume from CT or the TAVI procedure was unavailable or implausible, or if they had undergone prior valvuloplasty or had end-stage renal disease requiring dialysis.

### 2.3. Renal Function Assessment

Renal function was evaluated using serial measurements of serum creatinine (mg/dL) and estimated glomerular filtration rate (eGFR, mL/min/1.73 m^2^), obtained within 72 h before and within 72 h after the TAVI procedure. The eGFR was calculated using the Modification of Diet in Renal Disease (MDRD) equation [19].

Changes in renal function were quantified as the absolute difference between post-procedural and pre-procedural values.

(ΔCreatinine = post-TAVI creatinine − pre-TAVI creatinine;

ΔeGFR = post-TAVI eGFR − pre-TAVI eGFR).

In contrast to categorical classifications alone, renal function was primarily analyzed as a continuous variable to capture the full spectrum of both deterioration and recovery following TAVI.

Acute kidney injury (AKI) was defined according to Kidney Disease: Improving Global Outcomes (KDIGO) criteria as an increase in serum creatinine ≥0.3 mg/dL or ≥1.5 times baseline, assessed within the available 72 h post-procedural observation window [20,21,22]. Changes in eGFR were not employed to define AKI in accordance with established recommendations; rather, they were assessed as complementary continuous measures of renal function [23,24].

In order to investigate potential heterogeneity in renal response, patients were categorized into three groups based on eGFR: ≥60, 30–59, and <30 mL/min/1.73 m^2^, according to their baseline renal function.

In order to reflect bidirectional changes in renal function, patients were further stratified according to their post-procedural renal trajectory into those with a decrease versus no decrease in creatinine and those with an increase versus no increase in eGFR for descriptive analyses.

Additionally, the CKD-EPI equation was employed to compute eGFR as a sensitivity analysis, resulting in results that were consistent with the primary MDRD-based analysis [25,26,27].

In order to account for potential regression to the mean, baseline renal function was explicitly considered in all analyses, as outcomes were defined as change scores.

### 2.4. Statistical Analysis

Continuous variables were summarized using descriptive statistics including number of observations, mean, standard deviation, minimum, first quartile, median, third quartile, and maximum. Categorical variables were reported as absolute and relative frequencies. Distributional assumptions were evaluated using the Shapiro–Wilk test, with a *p*-value ≥ 0.10 indicating approximate normality. Between-group comparisons were performed using Student’s *t*-test for normally distributed variables and the Mann–Whitney U test otherwise. Associations between continuous variables and renal function changes were assessed using Spearman’s rank correlation coefficient, selected due to the absence of assumptions regarding linearity. Multivariable linear regression models were constructed to assess independent associations with Δcreatinine and ΔeGFR, including baseline renal function, age, sex, BMI, and ejection fraction while accounting for baseline renal function and potential confounding. Logistic regression was used to evaluate predictors of AKI. Comparisons across baseline renal function strata were performed using the Kruskal–Wallis test for continuous outcomes. Given the exploratory nature of the study, results were interpreted with emphasis on effect size rather than statistical significance alone.

Statistical significance was defined as a two-tailed *p*-value less than 0.05. All analyses were conducted using R statistical software, version 4.5.0 (R Foundation for Statistical Computing, Vienna, Austria).

## 3. Results

### 3.1. Study Cohort and Baseline Characteristics

After exclusion of implausible BMI values and missing data, 410 patients were included in the final analytic cohort. Mean age was 82.0 ± 8.7 years, and 46.9% were women. Baseline demographic, anthropometric, procedural, and renal characteristics are summarized in Table 1.

Mean baseline serum creatinine was 1.25 ± 0.79 mg/dL, and mean baseline eGFR was 58.0 ± 20.8 mL/min/1.73 m^2^. The cohort reflected typical elderly, multimorbid population with substantial baseline renal vulnerability.

Due to missing or incomplete data in some clinical records, the number of patients included in analyses of specific variables varies slightly. For example, weight, height, and BMI data were available for 363 patients, and accordingly, weight-adjusted contrast dose calculations were limited to this subset. Similarly, valve/annulus diameter and left ventricular ejection fraction data were available for 370 and 368 patients, respectively. Variables such as age, contrast volume, and serum creatinine were complete for all 410 patients. This variation reflects pairwise complete case analysis due to missing data in retrospective records, resulting in slightly different sample sizes across statistical tests.

### 3.2. Post-Interventional Renal Function Changes

**Figure 2:**
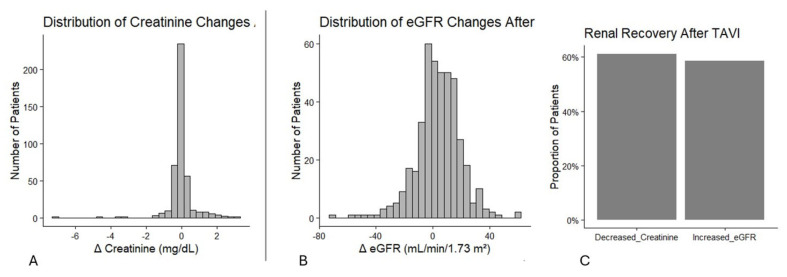
(**A**,**B**) Distribution of absolute changes in serum creatinine and eGFR within 72 h post-TAVI, demonstrating heterogeneous renal trajectories with a predominance of improvement. (**C**) Proportion of patients exhibiting renal recovery, defined as decreased creatinine or increased eGFR within 72 h following the procedure.

Renal function trajectories following TAVI were heterogeneous (Figure 2).

Among 410 patients with paired measurements, 63.3% showed decreased serum creatinine and 64.2% exhibited increased eGFR post-procedure. Median absolute changes were modest (ΔCreatinine −0.06 mg/dL; ΔeGFR +5 mL/min/1.73 m^2^), yet individual variability was substantial, ranging from marked decline to notable improvement. These results underscore renal recovery as a common early response after TAVI rather than a rare occurrence.

Spearman rank correlation analysis demonstrated generally weak associations between clinical or procedural variables and renal function changes. Weight-adjusted contrast volume showed the strongest relationship with renal outcomes, exhibiting a weak positive correlation with ΔCreatinine (ρ = 0.149, *p* = 0.005) and a weak inverse correlation with ΔeGFR (ρ = −0.148, *p* = 0.005), while all other variables—including age, BMI, valve diameter, and ejection fraction—were not significantly associated with renal changes (Table 2 and Table 3).

### 3.3. Associations with Renal Function Changes

Spearman correlation analyses identified weight-adjusted contrast dose as the parameter most consistently associated with renal function changes. A weak but statistically significant positive correlation was observed between weight-indexed contrast dose and ΔCreatinine (ρ = 0.149, *p* = 0.005), alongside an inverse correlation with ΔeGFR (ρ = −0.148, *p* = 0.005). Total contrast volume demonstrated only borderline associations with ΔCreatinine (ρ = 0.091, *p* = 0.079) and ΔeGFR (ρ = −0.094, *p* = 0.070). No meaningful correlations were observed for age, BMI, EF, or valve/annulus diameter.

Detailed correlation results are presented in Table 2 and Table 3, with additional exploratory analyses shown in Supplementary Figures S1–S6.

### 3.4. CKD Stratification

**Figure 3:**
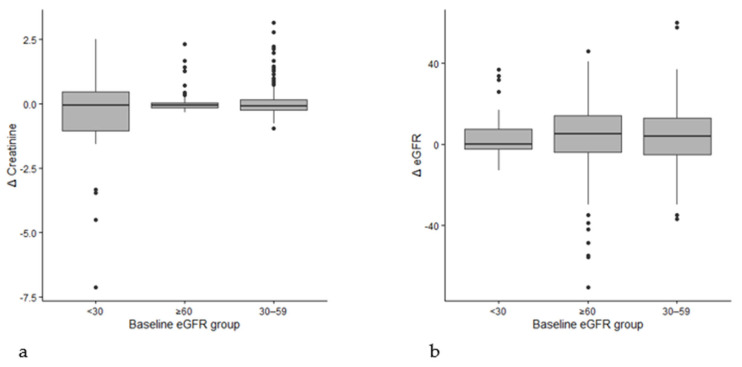
CKD stratification: (**a**) (Creatinine by baseline eGFR group) showing changes in serum creatinine stratified by baseline renal function categories (eGFR ≥60, 30–59, <30 mL/min/1.73 m^2^). Differences across groups were not statistically significant. (**b**) (eGFR by baseline group) illustrates changes in eGFR stratified by baseline renal function categories, demonstrating similar patterns of renal response across strata without significant group differences.

Renal function changes were further examined across baseline renal function strata (Figure 3).

The mean Δcreatinine varied numerically among the baseline eGFR groups (≥60: −0.002 mg/dL; 30–59: +0.063 mg/dL; <30: −0.433 mg/dL). However, these differences were not statistically significant (*p* = 0.321). The renal response patterns were comparable across groups, regardless of baseline kidney function, as evidenced by the fact that ΔeGFR did not differ (*p* = 0.598).

### 3.5. Multivariable Analysis

Δcreatinine was independently associated with baseline creatinine in multivariable linear regression analysis (β = −0.279, 95% CI −0.334 to −0.223, *p* < 0.001). Age, sex, and BMI were not significantly associated, whereas ejection fraction demonstrated a modest positive association (β = 0.012, *p* = 0.020). Although baseline eGFR exhibited a borderline inverse trend (β = −0.072, *p* = 0.057), no independent predictors were identified for ΔeGFR. Model fit was modest (adjusted R^2^ = 0.212 for creatinine; 0.008 for eGFR).

### 3.6. AKI

Acute kidney injury occurred in 57 patients (13.9%). In multivariable logistic regression analysis, no variable was independently associated with AKI, although baseline creatinine showed a borderline association (OR 1.20 per mg/dL, *p* = 0.082).

### 3.7. Sex Analysis

Sex-stratified analyses revealed a borderline difference in ΔCreatinine, with men showing a slightly greater median reduction compared with women (−0.08 vs. −0.03 mg/dL; *p* = 0.058).

No statistically significant sex-related difference was observed for ΔeGFR (*p* = 0.238). Overall renal response patterns were largely comparable between sexes, suggesting similar short-term renal adaptability following TAVI.

## 4. Discussion

In this retrospective cohort of patients who underwent transcatheter aortic valve implantation, a variety of formal findings are made that further complicate the current comprehension of post-procedural renal function changes. Initially, the short-term renal trajectories following TAVI were heterogeneous but frequently favorable. Within 72 h, over 60% of patients exhibited numerically improved renal markers, as evidenced by a decrease in serum creatinine and/or an increase in eGFR. Secondly, weight-indexed contrast exposure exhibited the most consistent, albeit weak, association with renal function changes among the examined demographic, anthropometric, and procedural variables. However, the observed correlations were of a low magnitude. These results, in conjunction with previous research that has demonstrated procedural and device neutrality, underscore the fact that renal recovery following TAVI is a common occurrence, despite the fact that it is underreported. It appears to be more influenced by patient-specific physiology than by prosthesis-related factors [18,28]. The observed correlations were modest (*p* ≈ 0.15), which restricted their immediate clinical applicability and supported their interpretation as hypothesis-generating.

These observations should be considered in the context of prior research that demonstrated that valve-related characteristics, such as prosthesis diameter and platform, were not independently associated with early renal outcomes or KDIGO-defined acute kidney injury. The two analyses, when considered in conjunction, offer complementary evidence. The present study emphasizes the significance of patient-specific and modifiable procedural variables, particularly contrast exposure, while device-related factors appear to be essentially neutral with respect to renal risk.

The significance of regression to the mean is underscored by the substantial impact of baseline creatinine on change scores in multivariable analysis, which is likely responsible for the apparent pattern of renal “improvement” following TAVI. When interpreting early post-procedural renal recovery, it is important to take this effect into account.

In older, multimorbid patients with limited physiological reserve or when guiding individualized procedural strategies, even modest associations between body-weight-normalized contrast dose and renal outcomes may have limited clinical relevance and should be interpreted cautiously due to the small effect sizes [29]. In patients at the extremes of body size, renal risk may not be completely captured by absolute contrast volume alone [30,31]. These results demonstrate a growing rationale for the integration of mL/kg-based contrast dosing into standard TAVI practice, in addition to established nephroprotective measures such as hydration protocols and the reduction in superfluous contrast exposure [32].

The perspective that early renal outcomes are primarily influenced by systemic and hemodynamic mechanisms rather than device-specific properties is further supported by the previously observed neutrality of valve-related factors. The significance of individualized procedural management over device selection is underscored by the association between weight-indexed contrast exposure and renal function alterations in this context.

A critical endpoint of this investigation is the enhancement of renal function. TAVI research has historically prioritized acute kidney injury through categorical definitions, thereby neglecting the bidirectional nature of renal alterations [33].

The high percentage of patients who have improved renal parameters indicates that reducing severe aortic stenosis may improve cardiac output, renal perfusion, and venous congestion [34]. These mechanisms may be particularly relevant in patients with cardiorenal interactions, who have renal dysfunction that is at least partially functional and reversible [35]. Recognizing renal recovery as a frequent early phenomenon following TAVI may assist in reorienting post-procedural renal assessment from an exclusively complications-centered perspective to a more balanced assessment of risk and benefit [36].

Sex-specific analyses revealed a minor, ambiguous difference in males who favored ΔCreatinine, while no significant sex difference was observed for ΔeGFR. Interpretation of this subtle indication should be approached with prudence. It may suggest that creatinine kinetics are influenced by sex-related variations in muscle mass, rather than genuine variations in glomerular filtration. This interpretation is corroborated by the absence of a corresponding variation in eGFR, which can partially account for sex. In general, renal outcomes were essentially comparable between women and men, suggesting that short-term renal adaptability after TAVI was equivalent.

The new findings corroborate previous publications regarding the vulnerability and variability of renal outcomes associated with TAVI. Valve intervention has been demonstrated to stabilize or enhance renal function, while contrast exposure has been associated with renal injury [3,12]. Nevertheless, the majority of previous research has focused on individual risk factors or utilized binary renal injury criteria [37,38]. By incorporating a diverse array of patient-specific and procedural variables and analyzing renal changes as a continuous outcome, this study provides a more thorough comprehension of the early renal dynamics following TAVI.

Certain limitations may necessitate specific considerations. The retrospective and exploratory design is inherently vulnerable to residual confounding, thereby impeding causal inference. The likelihood of a type I error was heightened due to the lack of correction for multiple comparisons and the slight variation in sample sizes across analyses. The evaluation of renal function was confined to the immediate post-procedural period, rendering the determination of long-term renal trajectories unfeasible. Furthermore, renal outcomes may have been influenced by unmeasured variables, including peri-procedural hemodynamics, drug exposure, and inflammatory markers. The cumulative contrast exposure may be influenced by the fact that the scheduling between pre-procedural CT imaging and the TAVI procedure was not standardized in this retrospective dataset and could not be systematically accounted for. Additionally, the combination of CT and procedural contrast was the primary metric for analyzing contrast exposure, and the separate effects of CT-derived versus procedural contrast were not specifically assessed. The effect magnitude and model stability were of particular concern, and the findings were interpreted with caution in light of the potential regression to the mean, as a result of the observational design and the use of change-score outcomes. The strength of inference is further restricted by the weak effect sizes and modest explanatory power of the models.

The findings are of significant practical importance, despite these limitations. They underscore the significance of a systematic approach to renal recovery and injury following TAVI, as well as weight-based contrast regimes. In order to verify these findings and determine which patients would benefit most from valve intervention, prospective studies with consistent nephroprotective protocols and extended follow-ups are required.

## 5. Conclusions

In this TAVI cohort, early renal function changes were common and frequently favorable; however, observed associations with clinical and procedural variables were weak. Baseline renal function emerged as the dominant determinant of change, highlighting the influence of regression to the mean. Weight-indexed contrast exposure showed a statistically significant but modest association with renal outcomes. Overall, these findings suggest limited independent predictive value of the examined variables and should be interpreted as hypothesis-generating.

## Figures and Tables

**Table 1:** Baseline Characteristics and Procedural Data. Variables include contrast volume, renal function, and echocardiographic measures. Descriptive statistics (mean, SD, quartiles, extremes) are reported. These values provide the reference framework for subsequent correlation and subgroup analyses regarding renal outcomes after TAVI.

Variable	n	Mean	SD	Min	Q1	Median	Q3	Max
Age (years)	410	82	8.7	61	78	82	85	100
CT Contrast (mL)	410	116.4	10.7	80	110	120	125	200
TAVI Contrast (mL)	410	231.0	65.5	50	200	211	250	500
Total Contrast (mL)	410	347.4	67.5	160	310	330	370	620
Weight-adjusted Contrast (mL/kg)	363	4.6	1.2	2	4	4	5	9
Valve/Annulus Diameter (mm)	370	27.8	3.6	20	26	29	29	34
Weight (kg)	363	78.1	14.3	44	70	76	85	160
Height (m)	363	1.698	0.089	1.48	1.65	1.70	1.75	1.97
BMI (kg/m^2^)	363	27.1	4.2	18	24.3	26	29.3	49.9
EF (%)	368	51.9	6.2	30	50	55	55	57
Creatinine before TAVI (mg/dL)	410	1.25	0.79	0.49	0.88	1.11	1.38	10.64
Creatinine after TAVI (mg/dL)	410	1.29	0.94	0.42	0.83	1.00	1.38	10.78
ΔCreatinine	410	0.034	0.539	−1.42	−0.20	−0.06	0.08	3.14
eGFR before TAVI (mL/min/1.73 m^2^)	410	58.0	20.8	5	41	58	71	138
eGFR after TAVI (mL/min/1.73 m^2^)	410	61.7	25.3	5	44	62	80	121
ΔeGFR	410	3.7	16.1	−71	−4	5	13	60

**Table 2:** Spearman Correlations with ΔCreatinine. Correlations between clinical/procedural factors and change in serum creatinine after TAVI. Correlations are expressed as Spearman’s rank coefficients (rS), a nonparametric measure of monotonic association. Reported *p* ^(1)^ values represent two-sided tests against the null hypothesis rS = 0. Weight-adjusted contrast demonstrated the strongest positive association, albeit weak (ρ = 0.149, *p* = 0.005).

Variable	n	rS	*p* ^(1)^
Age	410	0.057	0.277
BMI	362	−0.032	0.540
Total contrast (mL)	410	0.091	0.079
Weight-adjusted contrast (mL/kg)	362	0.149	0.005
Valve diameter (mm)	369	−0.027	0.602
EF (%)	367	0.026	0.613

rS = Spearman’s rank correlation coefficient. ^(1)^ *p*-value for test of null hypothesis rS = 0.

**Table 3:** Spearman Correlations with ΔeGFR: Spearman’s rank correlations (rS) between candidate factors and change in eGFR post-TAVI. The corresponding *p* ^(1)^ values test the null hypothesis that rS = 0. The only notable signal was a weak, inverse correlation with weight-adjusted contrast (ρ = −0.148, *p* = 0.005). Other variables, including age and EF, showed negligible associations.

Variable	n	rS	*p* ^(1)^
Age	410	−0.071	0.170
BMI	362	0.047	0.368
Total contrast (mL)	410	−0.094	0.070
Weight-adjusted contrast (mL/kg)	362	−0.148	0.005
Valve diameter (mm)	369	0.037	0.482
EF (%)	367	−0.004	0.935

## Data Availability

The data presented in this study are openly available in Pugliesi, Rosa Alba (2026). TAVI Dataset. figshare. Dataset. https://doi.org/10.6084/m9.figshare.31146352.

## Supplementary Materials

The following supporting information can be downloaded at: https://www.mdpi.com/article/10.3390/jcm15103726/s1. Figure S1. Age and Creatinine change after TAVI. Exploratory scatter plots assessing associations between age, left ventricular ejection fraction, and valve/annulus diameter with post-procedural renal function changes using Spearman correlation and LOESS smoothing. Figure S2. Age vs ΔeGFR. Scatter plot showing the association between patient age and post-procedural change in eGFR within 72 h after TAVI. Non-parametric LOESS smoothing and Spearman correlation illustrate the absence of a strong age-dependent renal effect. Figure S3. EF vs ΔCreatinine. Association between baseline left ventricular ejection fraction and short-term changes in serum creatinine after TAVI. Spearman correlation and LOESS smoothing demonstrate no meaningful relationship between systolic function and renal response. Figure S4. EF vs ΔeGFR. Scatter plot illustrating the relationship between baseline ejection fraction and post-TAVI eGFR changes. Exploratory non-parametric analysis indicates no clinically relevant association between cardiac systolic performance and renal recovery. Figure S5. Valve/Annulus Diameter vs ΔCreatinine. Relationship between valve/annulus diameter and post-procedural creatinine changes following TAVI. Spearman correlation and LOESS smoothing reveal no significant association between anatomical valve size and renal outcome. Figure S6. Valve/Annulus Diameter vs ΔeGFR. Scatter plot assessing the association between valve/annulus diameter and post-TAVI eGFR changes. Non-parametric exploratory analysis demonstrates minimal correlation between procedural valve size and renal function recovery.

## References

[1] Tödt J., Koul S., Yndigegn T., Angerås O., Bjursten H., Nozohoor S., Friberg Ö., Backes J., James S., Redfors B. (2026). Percutaneous and surgical management of aortic stenosis in the SWEDEHEART registry (2013–2023): A nationwide observational study. Lancet Reg. Health Eur..

[2] Seiffert M., Vonthein R., Baumgartner H., Borger M.A., Choi Y.-H., Falk V., Frey N., Hagendorff A., Hagl C., Hamm C. (2023). Transcatheter aortic valve implantation versus surgical aortic valve replacement in patients at low to intermediate surgical risk: Rationale and design of the randomised DEDICATE Trial. EuroIntervention.

[3] Abdelrahman A.A., Baraka M., Farag N., Mostafa A.E., Kamal D. (2025). Renal impairment in transcatheter aortic valve implantation: Incidence, predictors, and prognostic significance. BMC Cardiovasc. Disord..

[4] Steinke P., Akin I., Kuhn L., Bertsch T., Weidner K., Abumayyaleh M., Dudda J., Rusnak J., Jannesari M., Siegel F. (2025). The Prognostic Impact of Kidney Dysfunction in Unselected Patients Undergoing Coronary Angiography: In What Subgroups Does Kidney Dysfunction Matter?. J. Clin. Med..

[5] Jäckel M., Keller S., Prager E.P., Staudacher D.L., Schlett C., Zehender M., Bamberg F., Bode C., von Zur Mühlen C., Stachon P. (2023). The impact of transcatheter aortic valve implantation planning and procedure on acute and chronic renal failure. Cardiol. J..

[6] Venturi G., Pighi M., Pesarini G., Ferrero V., Lunardi M., Castaldi G., Setti M., Benini A., Scarsini R., Ribichini F.L. (2020). Contrast-Induced Acute Kidney Injury in Patients Undergoing TAVI Compared With Coronary Interventions. J. Am. Heart Assoc..

[7] Da Silva M.V.L., Nunes Filho A.C.B., Rosa V.E.E., Caixeta A., Lemos Neto P.A., Ribeiro H.B., Almeida B.O., Mariani J., Campos C.M., Abizaid A.A.C. (2021). Improvement of renal function after transcatheter aortic valve replacement in patients with chronic kidney disease. PLoS ONE.

[8] Belardi J.A., Albertal M. (2016). Acute kidney injury after TAVI: Predict, detect, and prevent. Catheter. Cardiovasc. Interv..

[9] Meche V., Kundnani N.R., Sharma A., Căpăstraru F.-M., Nistor D., Sarau C.A., Gaita L. (2024). Cardio-Renal Syndrome: Latest Developments in Device-Based Therapy. J. Clin. Med..

[10] Singh S., Pershad A. (2023). White paper on mitigating risk factors for acute kidney injury in TAVR: A protocol to decrease TAVR-associated AKI. Indian Heart J..

[11] Badran A.S., Gadelmawla A.F., Khelifa H., Hasanin E.H., Gbreel M.I. (2025). Evaluating Zero-Contrast Transcatheter Aortic Valve Implantation (TAVI) for Patients with Renal Impairment: A Pooled Meta-Analysis of 1505 Patients. Catheter. Cardiovasc. Interv..

[12] Itelman E., Awesat J., Codner P., Aviv Y., Grinberg T., Abitbol M., Shafir G., Skalsky K., Shechter A., Kornowski R. (2025). Effects of Contrast Media on Renal Function Following Computed Tomography Prior to Transcatheter Aortic Valve Implantation. J. Clin. Med..

[13] Schörghofer N., Knapitsch C., Hecke G., Clodi N., Brandstetter L., Cozowicz C., Hammerer M., Hergan K., Hoppe U.C., Scharinger B. (2025). TAVI Success Is More Than Just the Valve: CT-Derived Sarcopenia as a Major Determinant of Long-Term Survival. J. Cachexia Sarcopenia Muscle.

[14] Xu X., Zeng T., Chen S., Tian N., Zhang C., Chen Y., Deng S., Mao Z., Liao J., Zhang T. (2025). Acute kidney injury: Pathogenesis and therapeutic interventions. Mol. Biomed..

[15] Ko K., Zwetsloot P.-P., Voskuil M., Stella P., Leiner T., Kraaijeveld A. (2022). Clinically Significant Incidental Findings on CT Imaging During TAVI Work-up: A Systematic Review and Meta-Analysis. J. Invasive Cardiol..

[16] Doyle M.P., Woldendorp K., Ng M., Vallely M.P., Wilson M.K., Yan T.D., Bannon P.G. (2021). Minimally-invasive versus transcatheter aortic valve implantation: Systematic review with meta-analysis of propensity-matched studies. J. Thorac. Dis..

[17] Iannopollo G., Cocco M., Leone A., Saccà S., Mangino D., Picchi A., Reccia M.R., Fineschi M., Meliga E., Audo A. (2025). Transcatheter aortic-valve implantation with or without on-site cardiac surgery: The TRACS trial. Am. Heart J..

[18] Pugliesi R.A., Muna S.F., Mahken A.H., Maalouf N., Chatzis G., Apitzsch J. (2026). Procedural and Device Neutrality of Post-TAVI Renal Outcomes: A Multivariable Analysis of Valve Type, Size, and Anatomy. J. Clin. Med..

[19] Dajak M., Ignjatović S., Jovicić S., Majkić-Singh N. (2008). The values of estimated glomerular filtration rate calculated with creatinine and cystatin C based equations in healthy adults. Clin. Lab..

[20] Kellum J.A., Lameire N., Aspelin P., Barsoum R.S., Burdmann E.A., Goldstein S.L., Herzog C.A., Joannidis M., Kribben A., Levey A.S. (2012). Kidney Disease: Improving Global Outcomes (KDIGO) Acute Kidney Injury Work Group: KDIGO clinical practice guideline for acute kidney injury. Kidney Int. Suppl..

[21] Kappetein A.P., Head S.J., Généreux P., Piazza N., van Mieghem N.M., Blackstone E.H., Brott T.G., Cohen D.J., Cutlip D.E., van Es G.-A. (2012). Updated standardized endpoint definitions for transcatheter aortic valve implantation: The Valve Academic Research Consortium-2 consensus document. Eur. Heart J..

[22] Benaicha K., Aldroubi B., Yousuf P., Nath R., Saveeta F., Kanwal F., Fatima T., Hirani S. (2023). Factors Associated with Acute Kidney Injury in Patients Undergoing Transcatheter Aortic Valve Implantation: A Systematic Review and Meta-Analysis. Cureus.

[23] Li J., Lv J., Wong M.G., Shi S., Zan J., Monaghan H., Perkovic V., Zhang H. (2024). TESTING Study Biomarker Group. Correlation of Urinary Soluble CD163 Levels with Disease Activity and Treatment Response in IgA Nephropathy. Kidney Int. Rep..

[24] Généreux P., Piazza N., Alu M.C., Nazif T., Hahn R.T., Pibarot P., Bax J.J., Leipsic J.A., Blanke P., VARC-3 Writing Committee (2021). Valve Academic Research Consortium 3: Updated endpoint definitions for aortic valve clinical research. Eur. Heart J..

[25] Levey A.S., Bosch J.P., Lewis J.B., Greene T., Rogers N., Roth D. (1999). A more accurate method to estimate glomerular filtration rate from serum creatinine: A new prediction equation. Ann. Intern. Med..

[26] Nuis R.-J.M., Van Mieghem N.M., Tzikas A., Piazza N., Otten A.M., Cheng J., van Domburg R.T., Betjes M., Serruys P.W., de Jaegere P.P. (2011). Frequency, determinants, and prognostic effects of acute kidney injury and red blood cell transfusion in patients undergoing transcatheter aortic valve implantation. Catheter. Cardiovasc. Interv..

[27] Seiffert M., Schnabel R., Conradi L., Diemert P., Schirmer J., Koschyk D., Linder M., Kersten J.F., Grosser A., Wilde S. (2013). Predictors and outcomes after transcatheter aortic valve implantation using different approaches according to the valve academic research consortium definitions. Catheter. Cardiovasc. Interv..

[28] Kurmanaliyev A., Braukylienė R., Aldujeli A., Zhumagaliyev R., Aitaliyev S., Unikas R. (2025). Evaluating the Impacts of Procedural and Patient-Specific Factors on the Outcomes of Transcatheter Aortic Valve Implantation (TAVI). Medicina.

[29] Mach M., Hasan W., Andreas M., Winkler B., Weiss G., Adlbrecht C., Delle-Karth G., Grabenwöger M. (2020). Evaluating the Association between Contrast Medium Dosage and Acute Kidney Injury in Transcatheter Aortic Valve Replacement Using Different Predictive Models. J. Clin. Med..

[30] Mordhorst A., Yan T.D., Hoskins N., Gagnon J., Kazemi K. (2022). Percutaneous proximal axillary artery versus femoral artery access for endovascular interventions. J. Vasc. Surg..

[31] Torre D.E., Pirri C. (2025). Alternative Arterial Access in Veno-Arterial ECMO: The Role of the Axillary Artery. J. Clin. Med..

[32] Venturi G., Pighi M., Lunardi M., Mainardi A., Del Sole P.A., Tavella D., Setti M., Pesarini G., Benini A., Ferrero V. (2021). Contrast-Induced Nephropathy in Patients Undergoing Staged Versus Concomitant Transcatheter Aortic Valve Implantation and Coronary Procedures. J. Am. Heart Assoc..

[33] Yildirim S.E., Akar B., Palac B., Bozkurt H., Yildirim T., Kiris T., Avci E. (2025). Temporal Dynamics of the Association Between Acute Kidney Injury and Mortality After Transcatheter Aortic Valve Implantation: Insights from Time-Varying and Landmark Survival Analyses. J. Cardiovasc. Dev. Dis..

[34] Karamasis G.V., Kourek C., Alexopoulos D., Parissis J. (2025). Transcatheter Aortic Valve Implantation in Cardiogenic Shock: Current Evidence, Clinical Challenges, and Future Directions. J. Clin. Med..

[35] Hochstadt A., Avivi I., Ingbir M., Shacham Y., Merdler I., Granot Y., Viskin S., Rosso R., Banai S., Konigstein M. (2021). Clinically Significant High-Grade AV Block as a Reversible Cause for Acute Kidney Injury in Hospitalized Patients—A Propensity Score Matched Cohort. J. Clin. Med..

[36] Makki K., Ammar F.I., Fernandez J.A., AlGhamdi M.A., Alturkistani A.M., Hubayni R.A., Khahwry E.I. (2024). Incidence of Acute Kidney Injury Post Transcatheter Aortic Valve Implantation (TAVI): A Single-Center Experience. Cureus.

[37] Haase-Fielitz A., Altendeitering F., Iwers R., Sliziuk V., Barabasch S., Bannehr M., Hähnel V., Neuss M., Haase M., Apfelbacher C. (2020). Acute kidney injury may impede results after transcatheter aortic valve implantation. Clin. Kidney J..

[38] Wang J., Yu W., Zhou Y., Yang Y., Li C., Liu N., Hou X., Wang L. (2017). Independent Risk Factors Contributing to Acute Kidney Injury According to Updated Valve Academic Research Consortium-2 Criteria After Transcatheter Aortic Valve Implantation: A Meta-analysis and Meta-regression of 13 Studies. J. Cardiothorac. Vasc. Anesth..

