# Supplementary material for: Exploratory Analysis of Early Renal Function Changes After Transcatheter Aortic Valve Implantation (TAVI): Limited Predictive Value Beyond Baseline Renal Function
Source: J Clin Med. 2026 May 12;15(10):3726. doi: 10.3390/jcm15103726 (PMC13207590; doi:10.3390/jcm15103726)
Supplement: Supplementary file 1 [file jcm-15-03726-s001.zip › jcm-4273427-supplementary.pdf]

Supplementary Materials

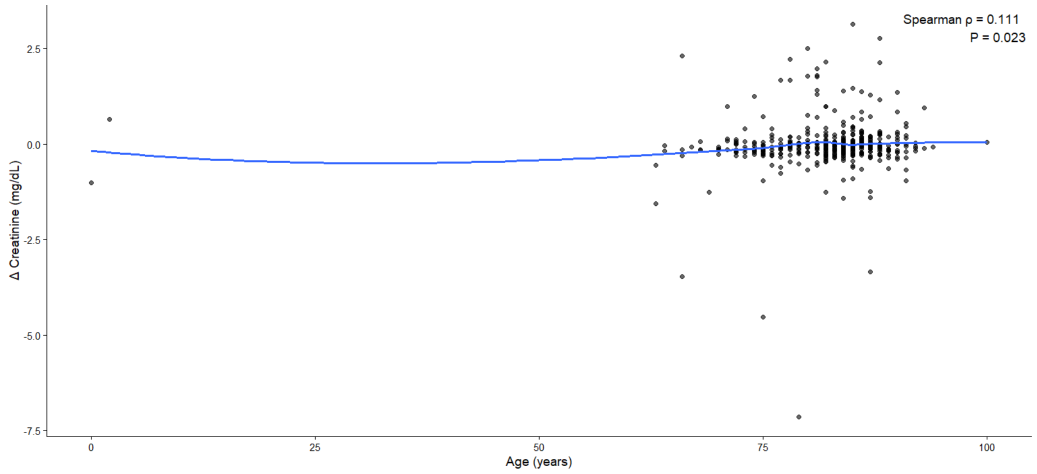

Figure S1. Age and Creatinine change after TAVI. Exploratory scatter plots assessing associations between age, left ventricular ejection fraction, and valve/annulus diameter with post-procedural renal function changes using Spearman correlation and LOESS smoothing.

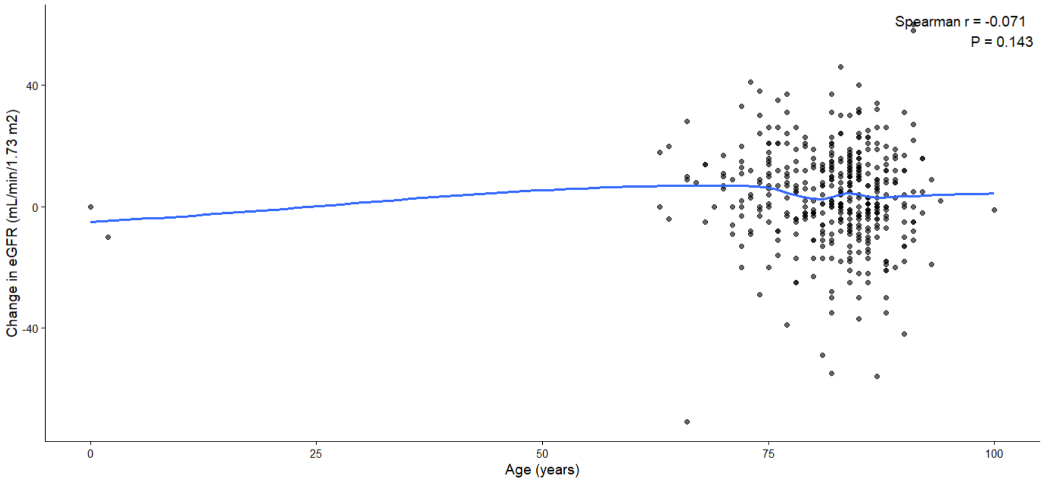

Figure S2. Age vs ΔeGFR**.** Scatter plot showing the association between patient age and post-procedural change in eGFR within 72 h after TAVI. Non-parametric LOESS smoothing and Spearman correlation illustrate the absence of a strong age-dependent renal effect.

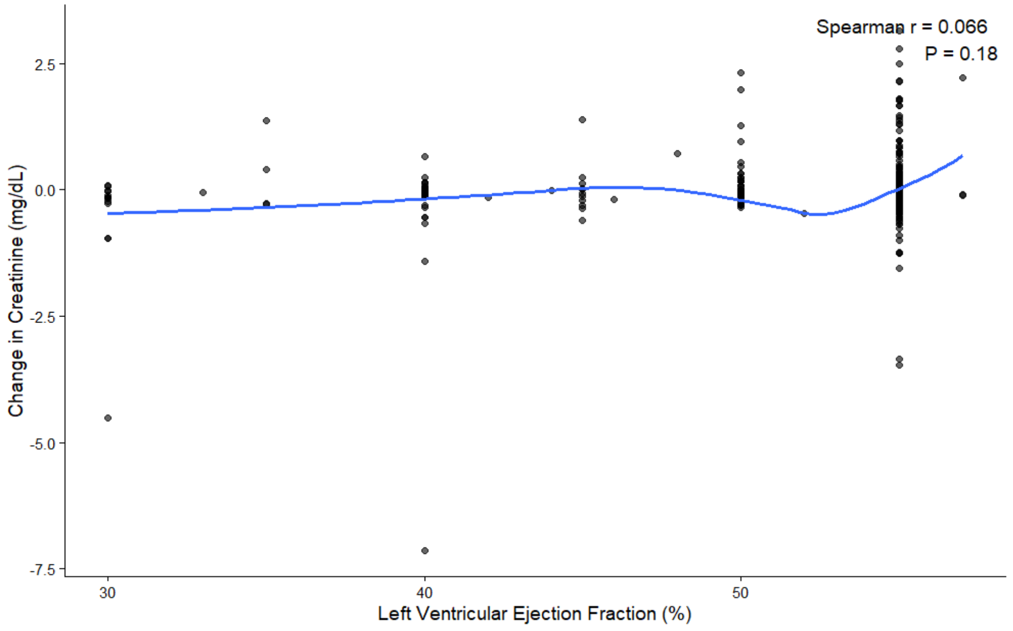

Figure S3. EF vs ΔCreatinine. Association between baseline left ventricular ejection fraction and short-term changes in serum creatinine after TAVI. Spearman correlation and LOESS smoothing demonstrate no meaningful relationship between systolic function and renal response.

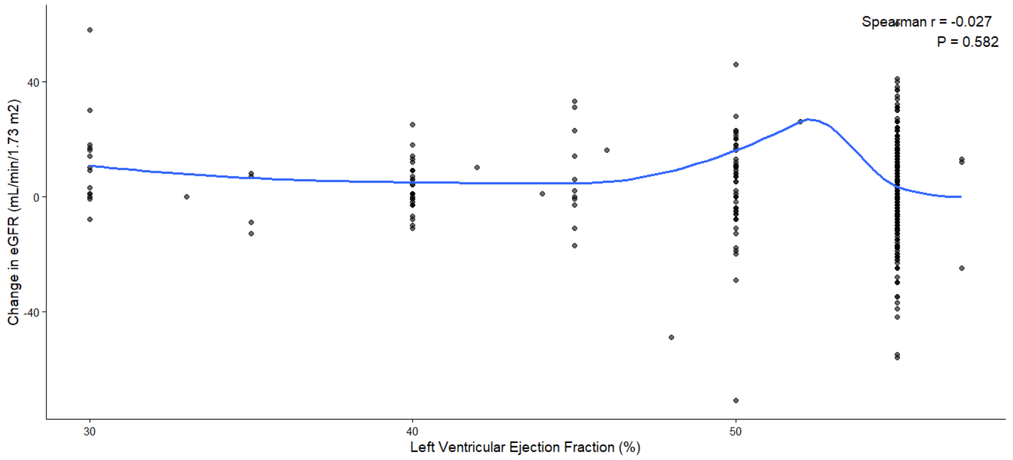

Figure S4. EF vs ΔeGFR. Scatter plot illustrating the relationship between baseline ejection fraction and post-TAVI eGFR changes. Exploratory non-parametric analysis indicates no clinically relevant association between cardiac systolic performance and renal recovery.

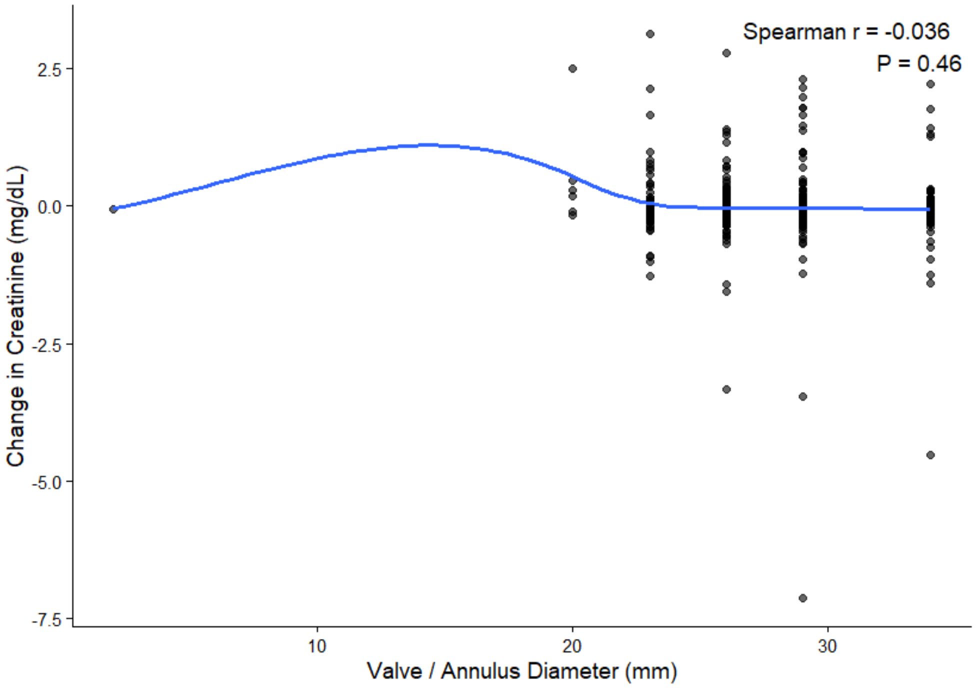

Figure S5. Valve/Annulus Diameter vs ΔCreatinine**.** Relationship between valve/annulus diameter and post-procedural creatinine changes following TAVI. Spearman correlation and LOESS smoothing reveal no significant association between anatomical valve size and renal outcome.

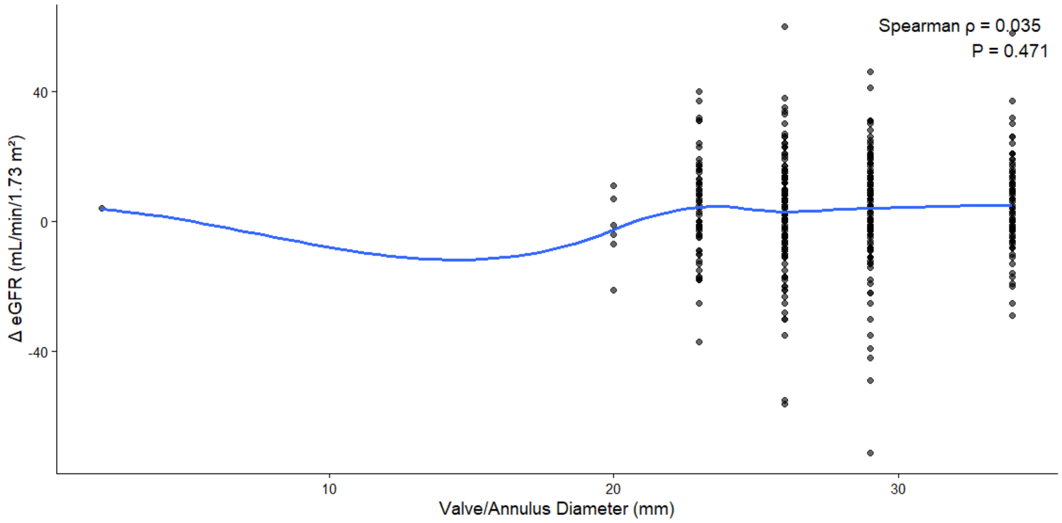

Figure S6. Valve/Annulus Diameter vs ΔeGFR. Scatter plot assessing the association between valve/annulus diameter and post-TAVI eGFR changes. Non-parametric exploratory analysis demonstrates minimal correlation between procedural valve size and renal function recovery.
